# Prospective memory evaluation in aging: new tools and methods

**DOI:** 10.1080/20009011.2017.1357413

**Published:** 2017-08-23

**Authors:** Mathieu Hainselin

**Affiliations:** ^a^ Centre de Recherche en Psychologie: Cognition, Psychisme et Organisations (CRP-CPO), EA 7273, Université de Picardie Jules Verne (UPJV), Amiens, France

Prospective memory (PM) is generally defined as ‘memory for actions to perform at a defined time in the future’, but recent publications have discussed this definition to specify it further [1]. PM is the most common cognitive complaint after 50 years of age, and everybody says to themselves once in a while, ‘I have something to do, but cannot remember what it is’ or ‘I should have done that before, why did I not remember at the appropriate moment?’

Twenty-seven years since the original paper by Einstein and McDaniel [2] on specific research in this area, there is still growing interest in PM (eight publications in 1994, 17 in 2004 and 103 in 2016, according to PubMed). The fields of experimental and clinical psychology, neuropsychology, medicine, neuroscience and education have developed different methods to explore and understand PM throughout life, in both healthy participants and patients. Beyond the classical debates concerning the PM age paradox [3] and the dissociation between prospective and retrospective components [4], this special issue highlights new methods to evaluate PM.

The first paper, by Blondelle et al. [5], is very informative on the role of regularity on PM throughout life. Moreover, this paper emphasizes the need to conduct an integrative and complete cognitive assessment, and not just to assess PM by itself.

The second paper, by Lecouvey et al. [6], uses a novel virtual reality method to assess PM within a virtual city. As well as allowing precise evaluation, the paradigm has a high level of ecological validity.

Future paradigms need to assess the role of binding, pointed out in both studies. Beyond the laboratory evaluations and scientific purposes, clinicians and researchers could develop new rehabilitation methods, including binding support to improve PM performance in everyday life.
